# Sociability modifies dogs’ sensitivity to biological motion of different social relevance

**DOI:** 10.1007/s10071-018-1160-8

**Published:** 2018-01-13

**Authors:** Yuko Ishikawa, Daniel Mills, Alexander Willmott, David Mullineaux, Kun Guo

**Affiliations:** 1 School of Life Sciences, University of Lincoln, Lincoln, LN6 7TS UK; 2 School of Sport and Exercise Science, University of Lincoln, Lincoln, LN6 7TS UK; 3 School of Psychology, University of Lincoln, Lincoln, LN6 7TS UK

**Keywords:** Biological motion, *Canis familiaris*, Sociability, Social relevance, Viewing perspective

## Abstract

Preferential attention to living creatures is believed to be an intrinsic capacity of the visual system of several species, with perception of biological motion often studied and, in humans, it correlates with social cognitive performance. Although domestic dogs are exceptionally attentive to human social cues, it is unknown whether their sociability is associated with sensitivity to conspecific and heterospecific biological motion cues of different social relevance. We recorded video clips of point-light displays depicting a human or dog walking in either frontal or lateral view. In a preferential looking paradigm, dogs spontaneously viewed 16 paired point-light displays showing combinations of normal/inverted (control condition), human/dog and frontal/lateral views. Overall, dogs looked significantly longer at frontal human point-light display versus the inverted control, probably due to its clearer social/biological relevance. Dogs’ sociability, assessed through owner-completed questionnaires, further revealed that low-sociability dogs preferred the lateral point-light display view, whereas high-sociability dogs preferred the frontal view. Clearly, dogs can recognize biological motion, but their preference is influenced by their sociability and the stimulus salience, implying biological motion perception may reflect aspects of dogs’ social cognition.

## Introduction

Biological motion, often created by point-light displays in which a few dots representing major joints of an otherwise invisible human/animal figure in action, can reveal characteristic motion patterns and enable naïve observers to infer an actor’s action (e.g. walking, dancing), gender, identity, intention and emotional states (Blake and Shiffrar 2007; Pavlova 2012). Sensitivity to biological motion emerges early in human perceptual development (e.g. infants show innate preference to look at biological over non-biological motion displays; Simion et al. 2008) and can be correlated with social cognitive performance (e.g. inferior biological motion detection ability in autistic children; Pavlova 2012); accordingly biological motion interpretation is believed to be an important way of optimizing adaptive behaviour efficiency and non-verbal communication in humans.

Sensitivity to conspecific and heterospecific biological motion has also been reported in many non-human species, such as chimpanzees (Tomonaga 2001), baboons (Parron et al. 2007), rhesus monkeys (Vangeneugden et al. 2010), common marmosets (Brown et al. 2010), dogs (Kovács et al. 2016), cats (Blake 1993), rats (MacKinnon et al. 2010) and chickens (Regolin et al. 2000). However, instead of measuring spontaneous viewing or approaching preference to biological motion presented just the once, the majority of studies have employed extensive training. For example, a review of the literature shows that after training chimpanzees, baboons and cats were capable of discriminating a point-light display portraying a quadrupedal walking conspecifics in lateral view from a control motion pattern (Blake 1993; Tomonaga 2001; Parron et al. 2007), whereas rhesus monkeys and rats could categorize human walking directions (MacKinnon et al. 2010; Vangeneugden et al. 2010). Common marmosets were also trained to remove a screen cover to view a walking hen animation or control movements (Brown et al. 2010). Other studies which have allowed repetitive viewing of similar point-light displays include those on chickens and dogs who were exposed to a similar biological motion pattern and a control for a prolonged period or on multiple occasions (Regolin et al. 2000; Kovács et al. 2016). This leads to a difficulty in determining whether the observed biological motion preference is an intrinsic capacity of the visual system or is shaped by reward-based associative learning.

In addition, it is not known whether biological motion preference in non-humans is associated with the social/biological relevance of the stimuli and/or the individual’s sociability. Biological motion representing different species or movement directions (e.g. a lateral view, moving tangentially to the viewer; or a frontal view, approaching the viewer) are of different levels of social relevance, since they represent different potentials for social interaction. Sociability, as a personality trait, is often defined in relation to an individual’s tendency to seek another’s company, but can also correlate with social cognitive performance (Gosling and John 1999; Fiske et al. 2007), such as, in humans, the ability to recognize face identities or positive facial expressions (Knyazev et al. 2008; Cheung et al. 2010). If biological motion perception is an inherent part of social cognition (Blake and Shiffrar 2007; Simion et al. 2008; Pavlova 2012), then it might be expected that its sensitivity in social animals will be modulated by both social relevance and sociability.

Among non-human species, dogs (*Canis familiaris*) are an ideal animal model to examine these issues. Pet dogs have frequent social interactions with other dogs and humans, are very attentive to human social and communicative cues (e.g. gaze/head direction and pointing gesture) (Hare and Tomasello 2005) and can visually differentiate an individual’s identity (Racca et al. 2010), facial expression and emotion (Racca et al. 2012; Müller et al. 2015; Albuquerque et al. 2016). So far, only one study has examined dogs’ sensitivity to repetitive human biological motion, and this involved repeated presentation and only a lateral view, although the authors reported a spontaneous looking preference towards upright point-light displays over scrambled or inverted ones (Kovács et al. 2016). This indicates dogs can “recognize” human biological motion cues, but other social factors related to biological motion recognition have not been explored.

Building upon previous biological motion research, we used a preferential looking paradigm without prior familiarization to examine dogs’ intrinsic visual sensitivity to both human and dog point-light displays from lateral and frontal views, and assess the influence of dogs’ sociability on their looking preferences. We predicted that dogs’ preferences would be influenced by the social relevance of the stimuli and the individual’s sociability.

## Materials and methods

### Subjects

Thirty-four healthy adult pet dogs (13 males, 21 females) of common breeds were recruited from the University of Lincoln dog database. All dogs were owned either by university staff members or local residents. The mean age was 4.8 ± 2.9 (mean ± SD) years; and breeds included 9 Labrador Retriever, 3 Golden Retriever, 3 Border Collie, 2 German Shorthair Pointer, 2 Jack Russell Terrier, 2 Miniature Schnauzer, 2 Siberian Husky, 2 Working Cocker Spaniel, 1 Border Terrier, 1 Malinois, 1 Nova Scotia Duck Tolling Retriever and 6 mixed breed dogs.

Ethical approval was granted by the ethical committee in the School of Life Sciences at the University of Lincoln. All procedures complied with the ethical guidance for the use of animals produced by the International Society for Applied Ethology.

## Materials

The walking human and dog point-light display videos were created from two women (26-year-olds) and two female dogs (one Labrador Retriever and one Welsh Springer Spaniel), whose motion was captured while walking on a treadmill at a constant speed of 2 km/h. Fifteen retroreflective markers were placed at the equivalent anatomical locations on both the humans and dogs: the anterior, left and right aspects of the head; and bilaterally on the top of the shoulder joint and on the lateral aspects of the elbow, wrist (paw for dogs), hip, knee and ankle joints. One additional marker was placed on the tail of the dog. The trajectories of the markers were tracked using ten Raptor motion capture cameras and Cortex software (Motion Analysis Corporation, Santa Rosa, CA) with 150 Hz sampling frequency. Each marker was reproduced as a point light in animations of the movement; a sixteenth marker was generated for the humans on the midline of the trunk at the estimated level of the belly button. Three 5-second point-light display video clips of different viewing perspectives with the resolution of 748 × 748 pixels were created for each model [1 frontal view, 2 (leftward and rightward) lateral views].

**Fig. 1:**
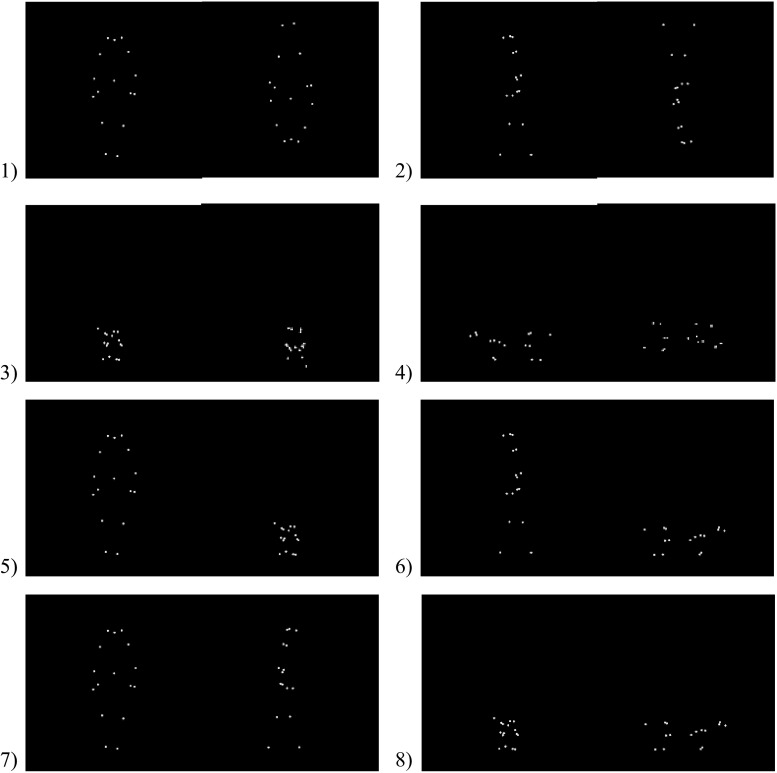
Examples of point-light display combinations: **1** frontal human upright versus frontal human inverted, **2** lateral human upright versus lateral human inverted, **3** frontal dog upright versus frontal dog inverted, **4** lateral dog upright versus lateral dog inverted, **5** frontal human upright versus frontal dog upright, **6** lateral human upright versus lateral dog upright, **7** frontal human upright versus lateral human upright, **8** frontal dog upright versus lateral dog upright

Previous studies on biological motion perception often use upside-down inverted point-light displays as control stimuli, and both human infants and non-human animals tend to look longer at an upright display when paired with its inverse (Blake and Shiffrar 2007; Simion et al. 2008; Pavlova 2012; Kovács et al. 2016). Therefore for each human and dog point-light display clip, we created an upside-down version to serve as the control condition. In total, 24 video clips, 4 models (2 humans + 2 dogs) × 3 views (1 frontal + 2 lateral) × 2 orientations (upright + inverted), were prepared for this study. These 24 clips were then paired with each other to form 16 trials consisting of 8 testing combinations (2 trials per combination, with different model subjects used in each trial): (1) frontal human upright versus frontal human inverted, (2) lateral human upright versus lateral human inverted, (3) frontal dog upright versus frontal dog inverted, (4) lateral dog upright versus lateral dog inverted, (5) frontal human upright versus frontal dog upright, (6) lateral human upright versus lateral dog upright, (7) frontal human upright versus lateral human upright, (8) frontal dog upright versus lateral dog upright (see Fig. 1 for examples). For each trial, the left/right position of the point-light display was randomized and counterbalanced across test dogs. The stimuli were presented in a pseudorandom order, ensuring no more than two consecutive trials showed the same combination.

### Experimental procedure

**Fig. 2:**
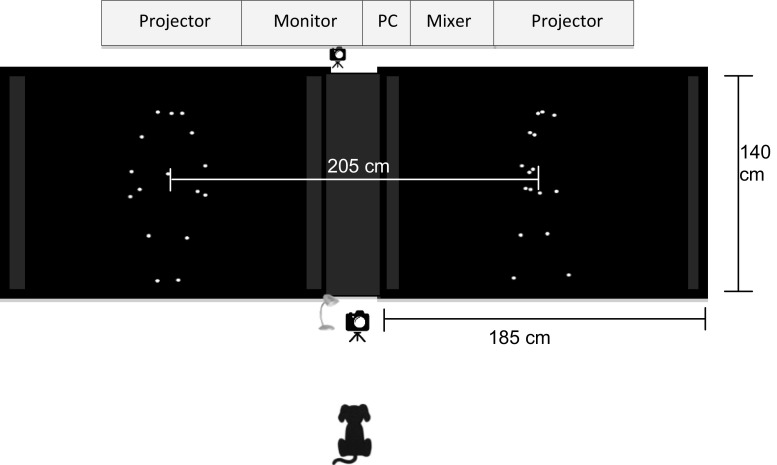
Experimental set-up and an example of testing combination (frontal human upright vs lateral human upright)

The experiment was conducted with a preferential looking protocol (Albuquerque et al. 2016) in a quiet, dimly lit room. During testing, dogs faced and stood in line with the middle of the two projection screens (185 cm × 140 cm), about 220 cm away (Fig. 2). One researcher (R1) monitored dog’s behaviour and controlled stimulus presentation, while another researcher (R2) stood behind the dog, with his/her hands on the dog’s shoulders. To avoid potential human interference, R2 was instructed to look down so he/she was unaware of the content of video presentation for a given trial.

The trial started by flashing an LED panel placed between the two projection screens to attract the dog’s attention. Once the dog’s gaze was oriented towards the middle, a pair of point-light displays was back-projected onto the screens for 5 s through two projectors (Optoma EX551, UK). The two displays were projected in the middle of each screen, separated by approximately 205 cm (~ 53°). The projected size of human and dog displays was up to 111 cm × 43 cm (~ 29° × 11°) and 49 cm × 84 cm (~ 13° × 22°), respectively.

During the presentation, the dog passively viewed the point-light displays. Its spontaneous looking behaviour (gaze/head direction) was recorded through a video camera (Sanyo CCD camera VVC-3312P, Japan) placed 150 cm away from the dog at the bottom of the LED panel. A second camera (Genie C8706/240, UK) was placed behind the projection screen to film the presented video clips. The outputs of two cameras were mixed (Panasonic WJ-MX 12 video mixer, Japan) and then displayed on a TV screen to allow R1 to monitor stimulus presentation as well as the dogs’ attention in real time.

All dogs completed 16 presentation trials. A short break between trials was provided if necessary. No reinforcement was given during this procedure, neither were the dogs trained on any other task with these stimuli. It was considered that with their lack of training, and in the absence of instrumental responding, their behaviour could be considered as spontaneous as possible.

Dog owners were also asked to complete a questionnaire about their dog, including details of gender, neuter status, age and sociability towards unknown dogs and humans. A 5-point Likert scale (5—strongly agree, 4—partly agree, 3—neutral, 2—partly disagree, and 1—strongly disagree) was used to rate six sociability questions, that my dog is “friendly towards unknown dogs”, “friendly towards unknown humans”, “aggressive towards unknown dogs”, “aggressive towards unknown humans”, “fearful towards unknown dogs” and “fearful towards unknown humans”.

In order to validate our subsequent classification of dogs into high- and low-sociability groups towards unknown humans or unknown dogs, we tested the prediction that the two groups should differ in their scores for relevant items within the more widely used Canine Behavioural Assessment and Research Questionnaire (C-BARQ) (Hsu and Serpell 2003). The C-BARQ items were distributed to owners several weeks after initial data collection. For each dog we calculated their “stranger-directed aggression” score, “stranger-directed fear” score and “dog-directed aggression/fear” score within C-BARQ. Mann–Whitney *U* tests were then used to test our prediction that the dogs classified into high- and low-sociability groups would show significant differences in the relevant C-BARQ scores.

### Experimental data analysis

All statistical analyses were undertaken using IBM SPSS v22. The recorded videos were off-line analysed frame-by-frame at 25 frames per second using BORIS version 2.993 (Friard and Gamba 2016); the direction of the dog’s gaze was classified as “left”, “right”, “central” and “out” (away from the screens in any direction). A second researcher independently coded videos from 10 dogs (~ 30% of the total data). Both coders were blind to the test conditions of all trials, and a good agreement between coders was found with intra-class correlation of 0.85 (95% confidence intervals = 0.81, 0.88).

A trial was considered as valid for further analysis only when the dog’s initial gaze was directed at the centre of the display at the beginning of point-light display presentation. Out of a total of 544 trials (34 dogs × 16 presentation trials), 432 trials (79%) met this requirement. For each valid trial, we calculated a preferential viewing percentage (PP) for each of the two presented point-light displays.

PP = the amount of time the dog looked at the left (or right) display divided by total looking time (left + right + centre) for the given trial.

After averaging individual dog’s PP for each testing combination, PPs across all dogs were compared. The normality of data for each testing combination was assessed using the Shapiro–Wilk test and revealed non-normal PP distributions (*p* > 0.05); therefore, Wilcoxon signed-ranks test was used throughout to compare two PPs for each testing combination.

When analysing the owner-completed questionnaires regarding their dogs’ sociability, the median ratings of “friendly”, “aggressive” and “fearful” towards unknown humans were 5.0, 1.0 and 1.0, whereas towards unknown dogs they were 4.0, 1.0 and 1.5, respectively. These skewed rating distributions meant it was inappropriate to compare separately these three sociability categories; accordingly dogs were divided into two similar-sized groups based on high or low composite sociability scores towards unknown humans and dogs, respectively. Specifically, nineteen dogs were grouped into the high human sociability group on the basis of them having a friendly score of 5, aggressive score of 1 or 2 and fearful score of 1 or 2 towards unknown humans. The remaining 15 dogs were allocated to the low human sociability group. Likewise, 20 dogs were grouped into the high dog sociability group with a friendly score of 4 or 5, an aggressive score of 1 or 2 and a fearful score of 1 or 2 towards unknown dogs, whereas the other 14 dogs were grouped into the low dog sociability group. To investigate whether biological motion preference was related to an individual’s sociability, PPs for each display combination were independently analysed for dogs in high- or low-sociability group towards unknown humans or dogs using the Wilcoxon signed-ranks test. A Mann–Whitney *U* test was applied for comparisons between the two sociability groups.

## Results

**Fig. 3:**
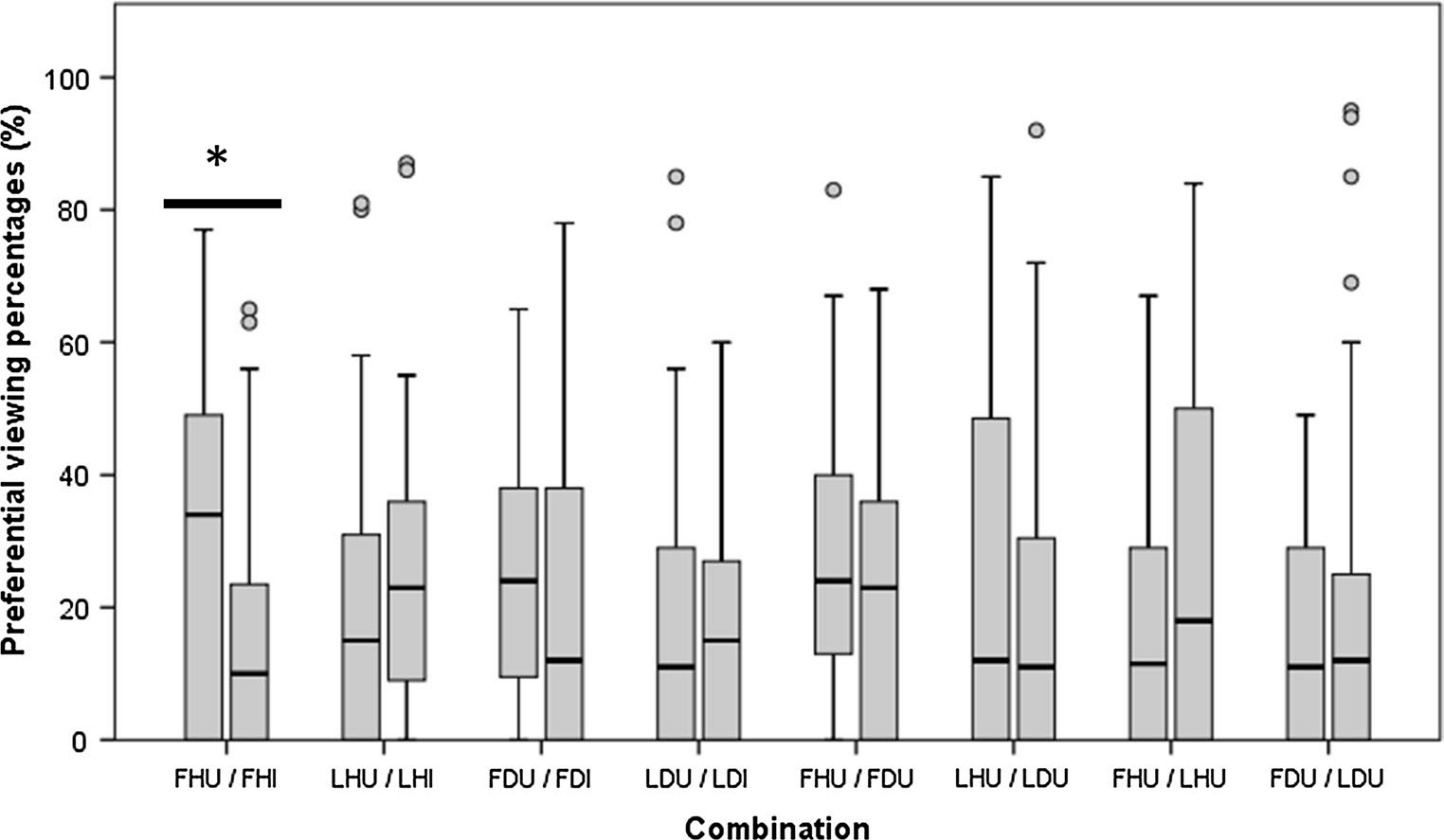
Minimum − maximum PP range (with circles as outliers) for two point-light displays in each testing combination. Lower, middle and upper lines in each box represent 25th, median and 75th percentiles of PP. *FHU* frontal human upright, *FHI* frontal human inverted, *LHU* lateral human upright, *LHI* lateral human inverted, *FDU* frontal dog upright, *FDI* frontal dog inverted, *LDU* lateral dog upright, *LDI* lateral dog inverted. *p < 0.05

Overall, dogs showed a clear viewing preference for biological motion depicting a frontal human view over its inverted control (34% median PP for frontal human upright vs 10% for frontal human inverted, *z*_32_ = − 2.06, *p* = 0.04; Fig. 3). No clear preference was observed for other human or dog forms of biological motion (lateral human upright, frontal dog upright, lateral dog upright) over control stimuli, nor between the two viewing perspectives from the same species (frontal human upright/lateral human upright, frontal dog upright/lateral dog upright), nor between the two species from the same viewing perspective (frontal human upright/frontal dog upright, lateral human upright/lateral dog upright) (*p* > 0.1).

Our classification of dogs into high- and low-sociability groups was validated by comparison with the relevant C-BARQ scores. There were significant differences in the combined relevant C-BARQ scores between the high- and low-sociability groups for both their reaction towards unknown humans (“stranger-directed aggression and fear” scores: Mann–Whitney *U* test, *u* = 33.0, *p* = 0.000, Cohen’s *d* = 1.37) and unknown dogs (“dog-directed aggression/fear” scores: *u* = 63.0, *p* = 0.007, Cohen’s *d* = 1.09). Furthermore, there were significant correlations between C-BARQ “stranger-directed aggression and fear” scores and our own dog sociability scores with unknown humans (Pearson Correlation, *r* = − 0.70, *p* = 0.000), and between C-BARQ “dog-directed aggression/fear” scores and our own dog sociability scores towards unknown dogs (*r* = − 0.60, *p* = 0.000), indicating lower C-BARQ score (less aggressive) was associated with higher sociability score (more sociable) in dogs.

When considering dogs’ sociability with unknown humans (Table 1A), dogs in low- and high-sociability groups showed comparably similar preference tendencies in their distribution of viewing time between the frontal human upright and frontal human inverted displays (34/16 vs 29/10%), but opposite preference tendencies in relation to the lateral human upright and lateral human inverted displays (24/13 vs 4/29%). Specifically, the high-sociability group looked significantly less at the lateral human upright display, and the viewing preference for the lateral human upright was significantly different between the two dog groups. It seems dogs prefer to look at frontal human views regardless of their sociability, but the lateral human view is preferred by less human-sociable dogs.

**Table 1:** Comparison between low and high-sociability groups towards humans (A) and dogs (B)

(A)	Low human sociability Median of PPs (*n*, *z*, *p*^a^)	High human sociability Median of PPs (*n*, *z*, *p*^a^)	Low versus high Human sociability *p* value^b^
FHU/FHI	34/16% (*n* = 14, *z* = − 1.71^c^, *p* = 0.09)	29/10% (*n* = 18, *z* = − 1.27^c^, *p* = 0.21)	0.17/0.70
LHU/LHI	24/13% (*n* = 15, *z* = − 1.03^c^, *p* = 0.30)	4/29% (*n* = 14, *z* = − 2.51^d^, *p* = **0.01**)	**0.01**/0.18
FHU/FDU	28/20% (*n* = 14, *z* = − 0.73^c^, *p* = 0.46)	24/23% (*n* = 19, *z* = − 0.44^c^, *p* = 0.66)	0.74/0.96
LHU/LDU	14/26% (*n* = 14, *z* = − 0.31^c^, *p* = 0.97)	4/7% (*n* = 18, *z* = 0.97^c^, *p* = 0.33)	0.33/0.14
FHU/LHU	15/28% (*n* = 14, *z* = − 1.13^d^, *p* = 0.26)	8/8% (*n* = 18, *z* = − 0.97^d^, *p* = 0.33)	0.54/0.16

(B)	Low dog sociability Median of PPs (*n*, *z*, *p*^a^)	High dog sociability Median of PPs (*n*, *z*, *p*^a^)	Low versus high Dog sociability *p* value^b^
FDU/FDI	17/29% (*n* = 13, *z* = − 0.16^d^, *p* = 0.88)	27/10% (*n* = 18, *z* = − 0.90^c^, *p* = 0.37)	0.42/0.44
LDU/LDI	29/6% (*n* = 13, *z* = − 1.88^c^, *p* = 0.06)	0/20% (*n* = 20, *z* = − 1.73^d^, *p* = 0.08)	**0.00/**0.18
FHU/FDU	31/27% (*n* = 13, *z* = 0.00^e^, *p* = 1.00)	22/14% (*n* = 20, *z* = 0.99^c^, *p* = 0.32)	0.54/0.37
LHU/LDU	26/27% (*n* = 13, *z* = − 0.55^c^, *p* = 0.58)	10/7% (*n* = 19, *z* = 0.65^c^, *p* = 0.52)	0.20/0.34
FDU/LDU	0/38% (*n* = 13, *z* = − 2.40^d^, *p* = **0.02**)	21/8% (*n* = 20, *z* = − 2.68^c^, p = **0.01**)	**0.00/0.03**

*p* values in bold indicate *p* < 0.05

*FHU* frontal human upright, *FHI* frontal human inverted, *LHU* lateral human upright, *LHI* lateral human inverted, *FDU* frontal dog upright, *FDI* frontal dog inverted, *LDU* lateral dog upright, *LDI* lateral dog inverted

^a^Two-tailed Wilcoxon signed-ranks test two-tailed, ^b^two-tailed Mann–Whitney *U* test between high- and low-sociability group for each point-light display, ^c^based on positive ranks, ^d^based on negative ranks, ^e^the sum of negative ranks equals the sum of positive ranks

Regarding relationships concerning the dogs’ sociability towards unknown dogs (Table 1B), the two groups showed the opposite tendency in their viewing time of lateral dog upright versus lateral dog inverted displays. Whereas low-sociability dogs tended to look longer at the lateral dog upright, high-sociability dogs preferred the lateral dog inverted, and the viewing preference for the lateral dog upright was significantly different between the two groups. Furthermore, when the frontal dog upright was paired with the lateral dog upright, the low-sociability group evidently preferred the lateral over frontal view, but the high-sociability group preferred the frontal over lateral view.

## Discussion

With a preferential looking paradigm, this is the first study to demonstrate in non-human animals a relevant sociability-modulated preference for biological motion of different species and viewing perspectives. Specifically, less sociable dogs preferred biological motion in a lateral view, whereas more sociable dogs preferred the frontal view, implying biological motion perception could potentially be a hallmark of social cognition and preferences in non-human social animals.

As biological motion processing is an intrinsic capacity of the human visual system (Blake and Shiffrar 2007; Simion et al. 2008; Pavlova 2012), and pet dogs often show human-like skills when processing social visual information (Hare and Tomasello 2005), such as faces (e.g. Guo et al. 2009), it is not surprising that they responded to point-light displays representing a walking human in frontal view by looking longer at it over its inverted control. A previous study examined only dogs’ responses to a walking human in lateral view and reported a preference over the inverted control (Kovács et al. 2016). By contrast, at a population level, we did not observe significant differences in viewing time between lateral human view and its control, or between frontal/lateral dog view and their controls. This may reflect a greater heterogeneity in sociability among the dogs used in our test compared to the previous study (Kovács et al. 2016). Given these former results (including loss of preference following oxytocin administration), this lack of preference should not be taken to mean that dogs cannot “recognize” these biological motion cues; as our analysis reveals, there are sociability-modulated individual differences in dogs’ sensitivity to the biological motion of different species and different directions. The preference for a frontal human view at the population level is likely due to its strong social/biological relevance. Humans are key social partners for pet dogs and frontal views of motion portray approaching actions associated with positive actions such as feeding and playing as well as negative actions such as possible threats, making such motion very salient regardless of their individual sociability.

By contrast, biological motion in a lateral view depicts humans/dogs passing by without an interactive intent, or the intention to avoid conflict with the viewer (active or mutual avoidance; Huntingford 2013; Riemer et al. 2013). This is of less relevance to sociable dogs but may be of interest to less sociable and potentially more socially vigilant dogs. When a lateral human/dog view was paired with its inverted control, high- and low-sociability dogs tended to look longer at the novel inverted movements and upright point-light displays, respectively. The potential outcome and thus significance of social interaction is different depending on an individual’s sociability. This is further supported by our finding that dogs with high- and low-sociability towards unknown dogs preferred the frontal and lateral view, respectively, since these two biological motion directions are related to different types of social experience and adaptive significance for the two groups as a result. For high-sociability dogs, interaction is an opportunity typically for a positive encounter, whereas for low-sociability dogs there is an increased risk of it being a negative experience and these animals are more likely to be more sensitive to the associated potentially negative cues (Harding et al. 2004).

Clearly, biological motion preference in dogs is modulated by stimulus social relevance and reflects individual differences in dogs’ sociability. Inferior biological motion performance (e.g. high (poor) detection threshold or low discrimination accuracy) is often correlated with impaired emotion perception in humans; so it has been suggested that biological motion sensitivity could be a reliable neuro-behavioural marker of human social cognition (Blake and Shiffrar 2007; Pavlova 2012). Our findings suggest this proposal might be extended to non-human social animals too, such as dogs. It should be noted that dogs’ sociability might be moderated by age, sex, environmental factors, individual experiences and genomic factors (e.g. Persson et al. 2016). Our current design and limited sample size from pet dogs does not allow us to disentangle these factors, but future research might usefully reveal the fuller role of these factors in biological motion preference.
